# Assessing the performance of index calibration survey methods to monitor populations of wide‐ranging low‐density carnivores

**DOI:** 10.1002/ece3.6065

**Published:** 2020-03-06

**Authors:** Egil Dröge, Scott Creel, Matthew S. Becker, Andrew J. Loveridge, Lara L. Sousa, David W. Macdonald

**Affiliations:** ^1^ Wildlife Conservation Research Unit Department of Zoology The Recanati‐Kaplan Centre University of Oxford Tubney UK; ^2^ Zambian Carnivore Programme Mfuwe Zambia; ^3^ Conservation Biology and Ecology Program Department of Ecology Montana State University Bozeman Montana; ^4^ Department of Wildlife Fish and Environmental Studies Umeå Sweden

**Keywords:** carnivores, IUCN criteria, lion, *Panthera leo*, population monitoring, spoor counts, statistical power, track counts

## Abstract

Apex carnivores are wide‐ranging, low‐density, hard to detect, and declining throughout most of their range, making population monitoring both critical and challenging. Rapid and inexpensive index calibration survey (ICS) methods have been developed to monitor large African carnivores. ICS methods assume constant detection probability and a predictable relationship between the index and the actual population of interest. The precision and utility of the resulting estimates from ICS methods have been questioned. We assessed the performance of one ICS method for large carnivores—track counts—with data from two long‐term studies of African lion populations. We conducted Monte Carlo simulation of intersections between transects (road segments) and lion movement paths (from GPS collar data) at varying survey intensities. Then, using the track count method we estimated population size and its confidence limits. We found that estimates either overstate precision or are too imprecise to be meaningful. Overstated precision stemmed from discarding the variance from population estimates when developing the method and from treating the conversion from tracks counts to population density as a back‐transformation, rather than applying the equation for the variance of a linear function. To effectively assess the status of species, the IUCN has set guidelines, and these should be integrated in survey designs. We propose reporting the half relative confidence interval width (HRCIW) as an easily calculable and interpretable measure of precision. We show that track counts do not adhere to IUCN criteria, and we argue that ICS methods for wide‐ranging low‐density species are unlikely to meet those criteria. Established, intensive methods lead to precise estimates, but some new approaches, like short, intensive, (spatial) capture–mark–recapture (CMR/SECR) studies, aided by camera trapping and/or genetic identification of individuals, hold promise. A handbook of best practices in monitoring populations of apex carnivores is strongly recommended.

## Graphical abstract

We tested and analyzed a popular rapid and cheap track survey method for large carnivores with randomly generated transects and empirical data on lion movements. We found that these methods are not able to reliably detect changes in populations of large carnivores often not even including the true population size in its confidence interval. We argue that the power of population estimates should meet criteria, preferably the IUCN guidelines, to detect population trends.

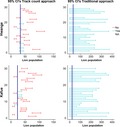

## 1 INTRODUCTION

Apex carnivores such as the African wild dog (*Lycaon pictus*), lion (*Panthera leo*), spotted hyaena (*Crocuta crocuta*), leopard (*Panthera pardus*), and cheetah (*Acinonyx jubatus*) have strong ecological, economic, and cultural importance in sub‐Saharan Africa. The wolf (*Canis lupus*), grizzly bear (*Ursus arctos*), and polar bear (*Ursus maritimus*) have similar importance in Europe and North America, as does the tiger (*Panthera tigris*) in Asia and the jaguar (*Panthera onca*) in the Americas. Most large carnivores have experienced substantial population declines and range contractions during the past two centuries (Ripple et al., 2014), mainly due to direct persecution or more indirectly through habitat conversion by a human population that increased about sevenfold, to more than 7 billion, with about 5.5 billion of those added in the past 90 years (Cohen, 2003, “Population Clock” 2019). Intact populations of apex carnivores are integral to ecosystem function and are an indication of ecosystem health (Ripple et al., 2014), as well as having economic and cultural value (Dickman, Macdonald, & Macdonald, 2011). Thus, there is an urgent need to conserve apex carnivores. They are, however, inherently difficult to conserve because they are conflict‐prone and require large, ecologically intact landscapes to support viable populations.

It is crucial that conservation and management decisions for large carnivores are based on reliable information (Jiménez et al., 2017). However, unbiased population or density estimates with sufficient precision to reliably detect trends are difficult to obtain for low‐density, wide‐ranging, and cryptic species that often occur in remote areas. Unbiased and precise estimates are best acquired through long‐term intensive studies that are often time‐consuming, expensive, and labor‐intensive (Beukes, Radloff, & Ferreira, 2017; Loveridge, Valeix, Davidson, Mtare, & Macdonald, 2016; Mweetwa et al., 2018). Long‐term studies allow the use of capture–mark–recapture (CMR) or spatially explicit capture–recapture (SECR) techniques, with individuals being identified by direct observation (Beukes et al., 2017; Elliot & Gopalaswamy, 2016; Loveridge, Valeix, Elliot, & Macdonald, 2016; Mweetwa et al., 2018) or camera trapping (Borah et al., 2013; Karanth, 1995; Kelly et al., 2008; Rich et al., 2019; Silver et al., 2004; Tarugara, Clegg, Gandiwa, & Muposhi, 2019). Genetic identification (Bellemain, Swenson, Tallmon, Brunberg, & Taberlet, 2005; Boersen et al., 2003; Kendall et al., 2009; Miotto, Cervini, Kajin, Begotti, & Galetti Jr, 2014; Solberg, Bellemain, Drageset, Taberlet, & Swenson, 2006; Spitzer, Norman, & Schneider, 2016) or a mix of these methods (Jiménez et al., 2017) can also be employed. In exceptional cases, distance sampling methods are employed where large carnivores are unusually visible, for example with polar bears (*Ursus maritimus*) (Aars et al., 2009; Stapleton, Atkinson, Hedman, & Garshelis, 2014) or carnivores on the shortgrass plains of the Serengeti (Durant et al., 2011).

In comparison with these methods, index calibration survey (ICS) methods are quicker and cheaper and have thus been used to describe population trends (Clevenger & Purroy, 1996; Mason & Macdonald, 1987; Smallwood & Fitzhugh, 1995). Managers need reliable population estimates or estimates with a consistent bias and reasonable precision to infer population trends, often quickly, while management and research budgets are small (Lindsey et al., 2018) and research capacity is limited. If surveys are intended to establish conservation priorities among sites, the bias of index methods must be consistent among the ecosystems being compared. Given these dilemmas, there is great interest in finding methods that can provide reliable population estimates across large areas with relatively minimal time and expense. For African carnivores, ICS methods, like track or “spoor” counts (and in the case of lions and hyaenas also call‐up/call‐in methods), have been widely adopted to play this role, particularly for lions.

Index calibration survey methods fundamentally rely on a predictable relationship between the index and the population size/density, as well as a constant detection probability of the index across the range of variables potentially affecting it (Anderson, 2003). Quantifying these relationships requires verification (Ericsson & Wallin, 1999; Graham, 2002; Stanley & Bart, 1991), and this has resulted in considerable debate on the use of ICS methods. Wilson and Delahay (2001) wrote that an ideal index is one in which varies consistently with changes in abundance of the target species with considerations given to factors that may influence index values even if abundance does not change. Anderson (2001) emphasized that ICS rests on critical but untested assumptions about detection probability, which is often assumed to be constant across habitats, observers, and characteristics of the target species. Anderson (2003) took this a step further and suggested that unless detection probabilities are estimated from the data, ICS studies provide “just numbers” that reveal little about abundance.

The challenges of employing ICS methods with large carnivores are well illustrated by the debate over trends in tiger (*Panthera tigris*) abundance in India. Traditionally censused with track count methods that assumed complete coverage and 100% detection, tiger estimates were criticized by Karanth et al. (2003) who suggested that changes in ICS estimates might correlate poorly with changes in actual density. Jhala, Qureshi, and Gopal (2011) defended the use of a combination of two indices, but Gopalaswamy, Delampady, Karanth, Kumar, and Macdonald (2015) found that imperfect detection, spatial heterogeneity in sampling, sampling uncertainties, and variation in true abundance all had strong effects on the accuracy of ICS methods. Furthermore, Gopalaswamy, Karanth, Delampady, and Stenseth (2019) show that estimates of trends in tiger populations are misleading because of the presence of high sampling‐based overdispersion and parameter covariance due to unexplained heterogeneity in detection probabilities. Such processes likely explain why field studies exhibit such a wide variation in degree of confidence in population estimates from ICS. In the latest countrywide tiger survey from India, there is still a lot of effort put into sign surveys, with over 500,000 km of walked transects, but the majority of the data contributing to the total estimates comes from CMR data from camera trapping (Jhala, Qureshi, & Nayak, 2019).

Despite the challenges encountered with carnivore population monitoring on other continents, in Africa, two different ICS techniques are commonly employed for a range of carnivore species and even recommended as the primary monitoring methods for lions (Funston & Henschel, 2018). While both techniques have received criticism (Belant et al., 2019; Rosenblatt et al., 2014; Whitman, 2006), both are in wide current use. One method commonly used to estimates populations of lions and hyaenas are call‐ups/call‐ins (Bauer, 2007; Begg, 2015, 2019; Belant et al., 2016; Brink, Smith, & Skinner, 2012, 2013; Cozzi, Broekhuis, Mcnutt, & Schmid, 2013; Ferreira & Funston, 2010, 2016; Groom, Funston, & Mandisodza, 2014; Midlane, Justin O'Riain, Balme, & Hunter, 2015; Ogutu & Dublin, 1998; Okot‐Omoya, Mudumba, Buckland, Mulondo, & Plumptre, 2013; Trinkel, 2009). In this method, audio lures (recordings of hyaena, lion, and distressed herbivores) are played through loudspeakers to attract lions and hyaenas. Because it is widely recognized that habituation and a range of other factors affect whether an animal is attracted and is surveyed (Brink, Smith, & Skinner, 2013; Mills, Juritz, & Zucchini, 2001; Ogutu & Dublin, 1998; Whitman, 2006), call‐ins require calibration with lions and hyenas to determine at what distances (and with what audio lure sounds) animals will respond. Furthermore, habituation can invalidate such calibrations, if animals reduce their response through time. To avoid this problem, track or spoor surveys are widely used to estimate population densities of many of the African large carnivores: lions, hyaenas, leopards, cheetahs, and African wild dogs (Bauer et al., 2014, 2015, 2017; Hanssen, Funston, Alfred, & Alfred, 2017; Funston et al., 2010, 2017; Groom & Watermeyer, 2019; Houser, Somers, & Boast, 2009; Midlane, Justin O'Riain, Balme, & Hunter, 2015; Stander, 1998a; Winterbach, Ferreira, Funston, & Somers, 2016). Stander (1998b) found a positive correlation between track frequency and independent estimates of population density for African wild dogs, leopards, and lions in northern Namibia. Funston et al. (2010) refined this method to account for substrate variation and concluded that “a combined model for all carnivore species on sandy soils served as a robust approach to predict large carnivore densities.” The regression model of Funston et al. (2010) included a nonzero intercept, which allows a track density of zero to predict a population density above zero. Winterbach et al. (2016) refined the model by dropping the nonzero intercept from the regression, but did not alter the way the uncertainty in the estimates were calculated; this methodology is hereafter what we refer to as the track count method.

In the track count method, transects, usually road based, are driven at a slow speed, in early morning when tracks of animals are most easily detectable, and the number of tracks of individual animals of several species and kilometers driven are recorded. Then, a track density is calculated as the number of tracks per 100 km driven. A constant, even across species, relationship between number of observed tracks of a species and actual density of the species in the area is assumed, and a density and/or population estimate is calculated from this relationship. The logic of the method is intuitively appealing: Carnivore tracks are more easily detected than carnivores themselves and can be rapidly collected over large areas; if track density can then be accurately converted to carnivore density and provide unbiased and precise estimates of population density, then this method could provide important information in an efficient way. Evaluating this premise is the focus of this paper.

Despite the promise of ICS methods such as track surveys for carnivore conservation, it remains essential that population estimates are paired with valid estimates of their uncertainty. For example, wildlife managers need measures of uncertainty to assess the likelihood that an apparent change in population size is real or to assess the likelihood that a population decline/increase of a given size (say 10%) would go undetected. We have concerns that current track survey methods overstate the precision of population estimates, for several reasons. First, these methods do not account for uncertainty in the original population estimates used to calibrate the relationship between track density and carnivore density. Second, variance in track density is converted to variance in carnivore density incorrectly; we refer to the discussion for a further explanation of this. We also assess variation in the number of encountered tracks between surveys, or an inconsistent detection probability, using a Monte Carlo simulation of theoretical transects and the movements of GPS‐collared animals. We use data from GPS‐collared lions from two populations (Hwange National Park in Zimbabwe and Kafue National Park in Zambia) to test how variation in track density from populations of known size is captured by population estimates using the track count method in a best possible scenario. In simulations comparing estimated and known population density, we test how variation in lion density and survey intensity affects the accuracy of the population estimate compared to known size population. Lastly, we discuss how issues with track surveys relate to the challenge of monitoring and conservation for large carnivores in general and lions in particular.

## 2 METHODS

Our simulations were designed to reflect a situation in which track surveys detected lions as well as possible. To compare the track count method between sites, we used data from two long‐term lion studies, one in the Northern part of Kafue National Park in Zambia and the other from the Northern and Eastern side of Hwange National Park in Zimbabwe, to allow comparison of results for simulations parameterized with data from areas with different lion and road densities. Our approach was to: (a) simulate, in R (R Core Team, 2018), track detection data for populations with movement trajectories based on GPS‐collared individuals, (b) use track count methods to produce an estimate of the population, and (c) compare the mean and confidence limits of the estimated population with the actual used population in each area. We began with data on the movements of GPS‐collared lions (see below) and the actual road network in the area within which they moved.

## 3 STUDY AREAS

Kafue National Park, 22,400 km^2^, is situated in western Zambia between 15°46′S 25°55′E. Mean annual rainfall ranges from 1,020 mm (in the North) to 530 mm (in the South), three perennial rivers, the Kafue, Lunga, and Lufupa run the length of the park (Midlane, Justin O'Riain, Balme, Robinson, & Hunter, 2014). Vegetation consists of Miombo and Kalahari woodland dominated by *Brachystegia* spp. and *Julbernardia* spp., munga and termitaria woodland dominated by *Acacia* spp., *Combretum* spp. and *Terminalia* spp., and munga scrub and grassland comprising open scrubland up to 3 m high and dambo, floodplain, and riverine grasslands (Midlane et al., 2015). The Kafue lion data from this study came from four neighboring lion prides in the northern section of the park. In Kafue National Park, lion was estimated at 1.83 lions per 100 km^2^ Midlane et al. (2015) with a 95% confidence interval of 0.86–2.80 based on track counts. However, a study currently in review, Vinks et al., estimates 3.43 lions per 100 km^2^ with a CI of 2.79–4.23 based on CMR.

Hwange National Park, 14,600 km^2^, is situated in north‐western Zimbabwe (19°00′S, 26°30′E). Mean annual rainfall is 600 mm and highly variable, and water is artificially supplied at water points in the dry season (Loveridge, Valeix, Davidson, et al., 2016). Vegetation consists of arid, dystrophic savannah forming a mosaic of *Combretum* sp., *Terminalia* sp., *Acacia* sp., and *Baikiaea* sp. communities on the Kalahari sands where this study was located (Chamaillé‐Jammes et al., 2006; Rogers, 1993). The Hwange lion data from this study came from five neighboring lion prides in the eastern part of the park. In Hwange, lion density was estimated at about 3.5 lions per 100 km^2^ (Loveridge, Valeix, Davidson, et al., 2016).

The actual size of the study areas was determined by the 100% minimum convex polygon of the used location points from the lions in the study. This led to a study area of 2,707 km^2^ in Kafue and 2,589 km^2^ in Hwange. The road network in Kafue was sparse with 467.2 km of roads contained within the study area. The road network in Hwange varied between sparse and locally dense, and the total length of roads within the study area in Kafue was 608.9 km.

### 3.1 Selection of sampled transects

From a mapped road network on each site, we randomly created sets of transects (10 km or 5 km) totaling ~20%, ~40%, ~60%, ~80%, and 100% of the total road network. These transect lengths are realistic and, with the set of sampling intensities just described, were long enough to ensure that simulations did not miss the entire home range of any lion and short enough to allow the efficient random selection of road segments to be included in transects. Appendix S1 provides a detailed description of transect selection. With these procedures, the exact sampling intensity was typically slightly less than the nominal intensity, so actual (rather than intended) transect lengths were used in all analyses. We added a buffer of 25 meters to all transects to increase the possibility of intersection with lion movement trajectories (see below) and decrease the possibility of missing intersections because of errors in the mapped location of either the roads or lion movement trajectories. We also report the penetration, as the amount of km^2^ of the study area per km driven, as reported in prior track surveys. A graphical diagram of the construction of transects can be found in Appendix S2.

### 3.2 Selection of lion movements and intersection with transects

We intersected the modeled transect network with lion movements from GPS‐collared lions in each study area. We constructed movement trajectories of sequential lion locations from the GPS collar data, which provided locations at intervals of 1–4 hr. We restricted the parameterization data to days for which we had data from at least one GPS‐collared lion in each lion group resident in the area. In Kafue National Park (where lion density was estimated to be relatively low, Midlane et al. (2015) estimated a density of 1.83 lions per 100 km^2^ and a CI of 0.86–2.80 based on track counts, a paper currently in review, Vinks et al., estimates 3.43 lions per 100 km^2^ with a CI of 2.79–4.23 based on CMR), we used data from one pride from 2016, two from 2017, and one from 2018. In Hwange (with slightly higher estimated lion density: (Loveridge, Valeix, Davidson, et al., 2016), we used data from four prides in 2011 and 2012 and a fifth from 2009 and 2010. We selected the lion movement trajectories for 24 hr prior to 6:30 a.m. on the day of survey and intersected it with the generated transects. Following this method, we intersected each randomly generated transect set with all days for which we had lion trajectories and thus simulated track surveys that started around sunrise when tracks are most visible.

In field applications, an attempt to avoid double‐counting of individuals, which cross roads multiple times in different nearby locations, is made by dismissing tracks if they are found within 500 m of another set of tracks of the same species and additional information like group composition, group size, and direction of movement indicates it might be the same set of animals (Funston & Henschel, 2018). As we did not incorporate group composition, size, and direction of movement of tracks, we used a proximity of 1,000 m (to further reduce the risk of double‐counting), to other tracks to dismiss a detection. We also simulated a scenario where all duplicated tracks, regardless of proximity to other tracks, were removed (scenario 3, see below). In our simulations, we assumed perfect detection whenever a lion trajectory intersected with a road transect, that all individuals present were detected by their tracks and that all lions in a group were present at all detections. In reality, lion prides sometimes move in subgroups, but fission–fusion dynamics do not alter the number of lions available for detection or the likelihood that their movements will intersect with a randomly selected survey segment. When a pride temporarily splits, we expect a decrease in the number of animals detected per detection that is offset by an increase in the number of detections. With respect to population estimation, these effects do not alter the number of lions available for detection, and any effect of pride fission–fusion on movement (if any exists) is embedded in the empirical data from GPS collars that we used to parameterize the model.

Using published methods, estimates from the track count method are considered reliable if 30 tracks, not clusters of tracks, are detected (Funston et al., 2010, Funston & Henschel, 2018). Given the densities of lions and roads in our two study areas, fewer than 30 tracks were detected in many simulated surveys with lower sampling intensities. To ensure a set of simulations in which enough tracks were detected, we also constructed surveys with a randomly selected transect set that was surveyed on 5 randomly selected days and combined the results. We did this 2,000 times and averaged the results. To mimic ideal circumstances with no double‐counting, we also analyzed the data with all duplicates removed (regardless of proximity to other detections) so that each pride could only be detected once a day. In the results, we name these scenarios as follows: (a) *regular surveys* (random transects, random day, duplicates of same pride within 1,000 m removed), (b) *replicated surveys* (random transects surveyed on 5 random days, duplicates of same pride on same day within 1,000 m removed), and (c) *replicated surveys with duplicates removed* (random transects surveyed on 5 random days, all duplicates of same pride on same day removed).

We created 400 sets of random transects for all intensities except for 100% intensity. At 100% intensity, nearly the whole road network is used, so that random transects cannot be generated. To determine the size of the study area and the roads to be considered for transects, we used lion GPS locations to create 100% minimum convex polygons with the AdehabitatHR package (Calenge, 2006) in R of each pride and then combined the polygons. We only used locations from the dates included in the construction of lion movement trajectories (see below). The area of this combined minimum convex polygon was considered the size of the study area, and we clipped the sampled road network to fall within this polygon. Density depends on the size of the area estimated to be surveyed, and we mainly calculate it here as densities are more easily compared to other areas and the most commonly reported population parameter.

### 3.3 Kafue lion data

From Kafue National Park, we used data from four neighboring prides, which consisted of three, five, five, and 10 subadult and adult lions, respectively. These prides were fitted with Telonics GPS satellite collars, fitted by a licensed Zambian veterinarian, with permits and protocols approved by the Department of National Parks and Wildlife. As there was no complete overlap in dates between all collars (one in 2016, two from 2017, and one from 2018), we used the day of year, irrespective of which year the data were collected. For 114 days of year, we had movement trajectories of all four prides, and we assumed the pride size to be constant over all these days. Thus with 400 different transect sets for each survey intensity, 45,600 surveys were simulated at each survey intensity.

The combined polygon for the Kafue prides was 2,707 km^2^, yielding a density of 0.85 lions per 100 km^2^. This density is not equivalent to the actual density of lions in Kafue, because the simulated population does not include other lions whose movements were not monitored with GPS collars; the simulation just investigates the relationship between track counts and density for these four prides with complete data on movements. The maximum survey intensity in Kafue was 5.86 km of road surveyed per km^2^ (467.2 km of road in a 2,707 km^2^ study area) of the study area for regular surveys where transects were surveyed once and 1.16 for replicated surveys where transects were surveyed five times.

### 3.4 Hwange lion data

Road and lion data from Hwange were treated the same as those from Kafue, using the combined 100% minimum convex polygons of all lion groups to select the study area and to define the extent of the sampled road network. Transects were built at the same intensities as in Kafue. We deleted some roads in very high road density areas around tourist camps and dead‐end spur roads shorter than 5 km, as these made the random selection of roads challenging and did not alter inferences. Movement data from five neighboring prides were used. These prides were fitted with GPS satellite collars from Africa Wildlife Tracking, Pretoria, South Africa, fitted by staff trained, and certified by the Wildlife Group of the Zimbabwean Veterinary Association and the Wildlife Unit of the Government Veterinary Services. This took place under animal handling protocols following the “Code of Practice for Biologists using Animals” from the Department of Zoology at the University of Oxford and approved by University of Oxford, Biomedical Sciences, Animal Welfare and Ethical Review Body (AWERB). Four prides yielded GPS data which coincided in time in 2011 and 2012, with the fifth pride yielding GPS data from 2009 and 2010. These prides numbered two, three, six, eight, and 14 subadult and adult lions. For 130 days of year, we had movement trajectories of all five prides, and we assumed the pride size to be constant over all these days. Thus, with 400 different transect sets for each survey intensity, 52,000 surveys were simulated at each survey intensity.

In Hwange, the combined minimum convex polygons for the lion prides was 2,589 km^2^, providing a density of 1.53 lions per 100 km^2^. Again, this density is not to be mistaken for the actual density of lions in Hwange; these were the lion prides included in our simulation to investigate the relationship between track counts and density for these five prides with complete data on movements. The total road network available for surveying was 608.9 km; this was slightly reduced in the 100% intensity surveys to 592.5 km, as the road network was divided into segments of up to 10 km but segments shorter than 500 m were discarded. Thus, the maximum intensity possible in Hwange was 4.37 km of road surveyed per km^2^ study are for surveys where transects were surveyed once and 0.87 where transects were surveyed five times.

### 3.5 Assessing model performance

From the recorded intersections between transects and lion movement trajectories from a population where all prides and individuals were known, we calculated the number of tracks recorded per transect, per survey (One survey is a complete set of transects for the particular survey intensity on a single day or 5 separate days) and calculated the mean and variance track density using the same methods as recent track surveys in real lion populations. Population estimates were calculated using the method developed by Winterbach et al. (2016) using the following formula to estimate the populations of lions and other large African carnivores: observed track density = 3.26 × carnivore density where track density is expressed in units of tracks per 100 km driven, and carnivore (lion) density is expressed in individuals (lions) per 100 km^2^. Following published methods for track counts, confidence intervals on population density were calculated with the coefficient of variance (CV) using two methods. The first method was developed by Funston et al. (2010). They found that this CV of observed tracks was independent of lion density and soil type and developed the following formula based on regression to estimate the CV in observed tracks: $CV\left(dt-t\right)=58.33n_{i}^{-0.36}$ where d*t* − *t* is the distance between observed tracks, and *n_i_* is the number of observed tracks, and we refer to this as the track count CV approach. The second method calculated the CV as the ratio of the standard deviation (*s*) of tracks per transect to the mean of tracks per transect (*N*). We refer to this as the traditional CV approach (also used by Bauer et al. (2017)), because it mimics the standard statistical formula $CV=\sqrt{\frac{s^{2}}{N}}=\frac{s}{\sqrt{N}}$. We emphasize, however, that the denominator of the traditional CV approach inadvertently substitutes *N* where *√N* is required, thus overstating the precision of population estimates.

Because we were primarily interested in testing the bias and precision of these method, we report (a) the percentage of cases in which the calculated confidence intervals for each method captured the true population size and (b) half the width of confidence intervals compared to the estimate (half relative confidence interval width) as a measure of power to detect population trends. We emphasize that for these simulations, the true lion population was known (the total number of lions in the prides from which we used locational data), there was perfect detection when lion movement trajectories and transects intersected, and the study area and thus the sampled transects were exactly matched to the area used by those prides during the time of study. A combination of the accuracy (percentage of times the true density was captured in the calculated confidence interval) and precision provided insight about the power with which these methods can detect changes in a lion population, or populations of other wide‐ranging, low‐density species, and how useful they are for this. To measure precision, we calculated the half relative confidence interval width (HRCIW) by:

$$HRCIW=\frac{0.5\times \left(UCL-LCL\right)}{\hat{N}}\times 100$$

where UCL and LCL are the upper and lower confidence limits, and *N̂* is the population estimate (which can be replaced with a density estimate). This HRCIW provides an easily interpretable measure of the magnitude of population change that must occur for it to be detectable with specified confidence.

## 4 RESULTS

The detailed results of regular surveys, without replication, can be found in Table 1. The minimum, mean, and maximum values represent those values from all simulations for the particular site and survey intensity. In Kafue, with regular surveys with between 20% and 100% of the roads surveyed, mean track density varied between 4.8 tracks per 100 km (at 100%) to 5.8 (at 20%), with the difference (width) between minimum and maximum track density, ranging from 14.72 (at 100%) to 61.80 (at 20%). In surveys where 100% of the road network was included, 1.8% of surveys intersected no tracks (Table 1).

**Table 1:** Results from regular surveys (random transects, random day, duplicates of same pride within 1,000 m removed)

Site	% of roads surveyed	Average survey length (km)	Average penetration (km^2^ per km driven)	Min track density (tracks/100 km)	Mean track density (tracks/100 km)	Max track density (tracks/100 km)	% of surveys with 0 tracks detected
Kafue	20	88.9	30.45	0	5.8	61.80	48.6%
Kafue	40	177.8	15.22	0	5.57	39.45	21.6%
Kafue	60	276.5	9.79	0	5.35	27.12	8.6%
Kafue	80	371.6	7.28	0	4.93	21.06	4.0%
Kafue	100	462.1	5.86	0	4.82	14.72	1.8%
Hwange	20	118.1	21.92	0	7.38	56.80	37.1%
Hwange	40	236.2	10.96	0	7.30	38.09	16.0%
Hwange	60	354.3	7.31	0	6.84	28.25	9.9%
Hwange	80	477.7	5.42	0	5.91	20.90	7.7%
Hwange	100	592.5	4.37	0	5.59	15.19	6.9%

In Hwange, with regular surveys with between 20% and 100% of the roads surveyed, mean track density varied between 5.59 (at 100%) to 7.38 (at 20%) tracks per 100 km, with the width of track density ranging from 15.19 (at 100%) to 56.80 (at 20%). In surveys where 100% of the road network was included, 6.9% of surveys intersected no tracks (Table 1).

The detailed results from replicated surveys can be found in Table 2. In Kafue, in replicated surveys with between 20% and 100% of the roads surveyed had a mean track density ranging from 4.85 to 5.88, with the width of track density ranging from 7.49 (at 100%) to 22.71 (at 20%). For survey intensities above 20%, there were no instances in which no tracks intersected with surveyed transects (Table 2).

**Table 2:** Results from replicated surveys (random transects surveyed on 5 random days, duplicates of same pride on same day within 1,000 m removed

Site	% of roads surveyed (5 times)	Average survey length (km)	Average penetration (km^2^ per km driven)	Min track density (tracks/100 km)	Mean track density (tracks/100 km)	Max track density (tracks/100 km)	% of surveys with 0 tracks detected
Kafue	20	444.6	6.09	0	5.88	22.71	13.0%
Kafue	40	888.9	3.05	0.34	5.60	14.17	0%
Kafue	60	1,382.3	1.96	0.94	5.36	12.29	0%
Kafue	80	1,857.9	1.46	1.41	5.64	14.43	0%
Kafue	100	2,310.5	1.17	1.69	4.85	9.18	0%
Hwange	20	590.4	3.65	0	7.31	23.02	2.8%
Hwange	40	1,180.9	1.83	0.85	7.45	17.72	0%
Hwange	60	1,771.5	1.21	1.58	6.83	17.16	0%
Hwange	80	2,388.3	0.90	1.73	6.72	14.70	0%
Hwange	100	2,962.6	0.73	1.35	5.61	11.34	0%

In Hwange in replicated surveys with between 20% and 100% of the roads surveyed, the mean track density varied between 5.61 (at 100%) and 7.45 (at 40%), with the width of track density ranging from 9.99 (at 100%) to 23.02 (at 20%). For sampling intensities above 20%, there were no instances in which no tracks intersected with surveyed transects (Table 2).

The detailed results from replicated surveys, with duplicates removed, can be found in Table 3. In Kafue in replicated surveys with duplicates removed with 20%–100% of the roads surveyed, the mean track density varied between 2.93 (at 100%) to 4.50 (at 20%), with the width of track density ranging from 2.7 (at 100%) to 13.32 (at 20%). For survey intensities above 20%, there were no instances in which no tracks intersected with surveyed transects (Table 3). In Hwange in replicated surveys with duplicates removed with between 20% and 100% of the roads surveyed, mean track density varied between 2.79 (at 100%) to 5.51 (at 20%), with the width of track density ranging from 3.28 (at 100%) to 13.66 (at 20%). For sampling intensities above 20%, there were no instances in which no tracks intersected with surveyed transects (Table 3).

**Table 3:** Results from replicated surveys with duplicates removed (random transects surveyed on 5 random days, all duplicates of same pride on same day removed

Site	% of roads surveyed (5 times)	Average survey length (km)	Average penetration (km^2^ per km driven)	Min track density (tracks/100 km)	Mean track density (tracks/100 km)	Max track density (tracks/100 km)	% of surveys with 0 tracks detected
Kafue	20	450.4	6.01	0	4.50	13.32	2.6%
Kafue	40	900.1	3.01	0.33	4.12	7.94	0%
Kafue	60	1,399.5	1.93	0.57	3.73	6.87	0%
Kafue	80	1,993.5	1.36	1.01	3.01	4.98	0%
Kafue	100	2,329.7	1.16	1.46	2.93	4.16	0%
Hwange	20	597.4	3.61	0	5.51	13.66	0.6%
Hwange	40	1,195.2	1.80	0.59	4.84	8.75	0%
Hwange	60	1,790.5	1.20	0.79	4.00	6.92	0%
Hwange	80	2,545.6	0.85	0.85	3.06	4.68	0%
Hwange	100	2,969.8	0.73	0.64	2.79	3.92	0%

**Figure 1:**
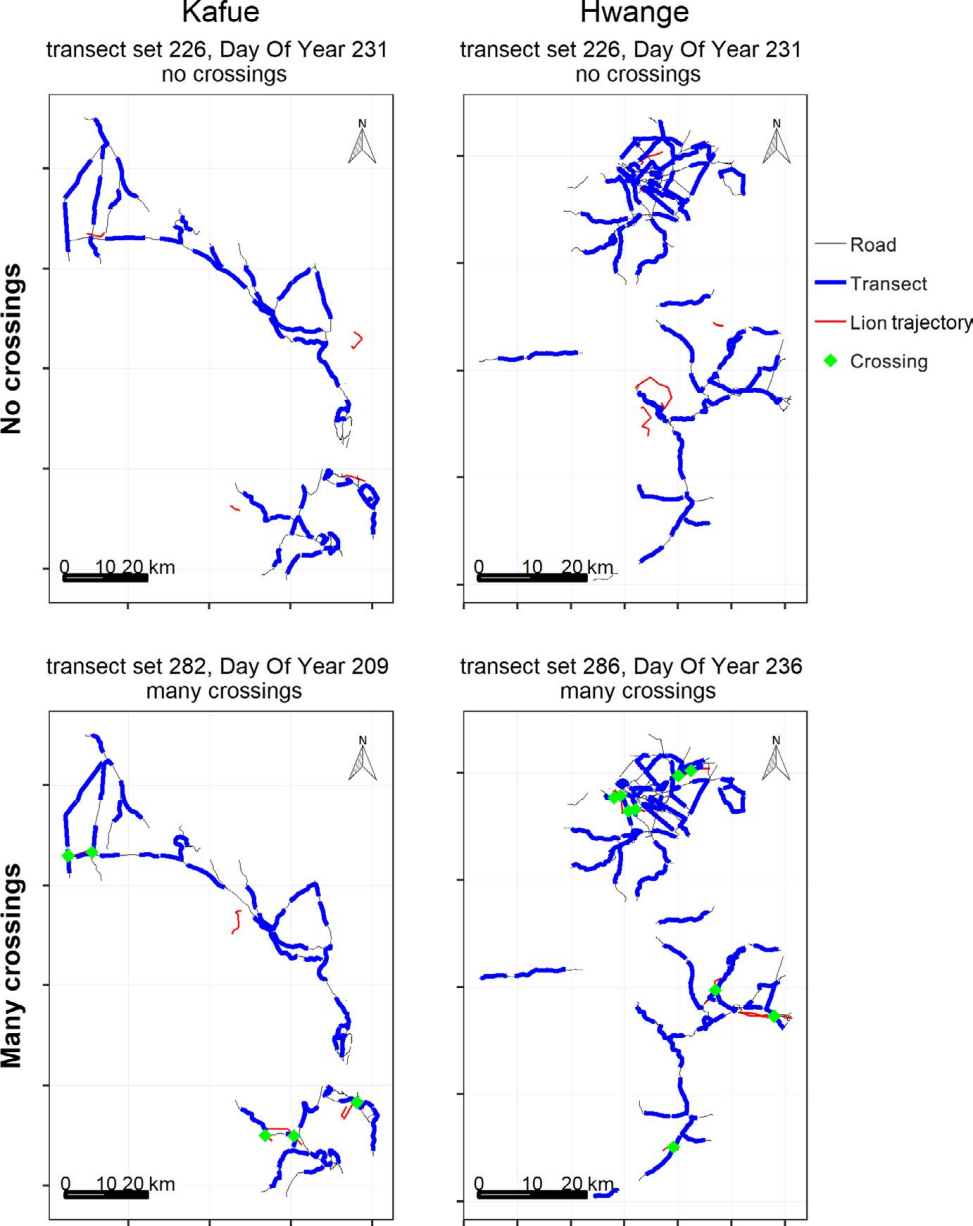
Scenarios where no lion tracks were detected and scenarios where many lion tracks were detected, both at 60% survey intensity, which represents a penetration rate of 9.79 for Kafue and 7.31 for Hwange

Figure 1 shows examples of regular surveys in each ecosystem, including the road network, the sampled transects at 60% survey intensity, and lion movement trajectories for the 24 hr preceding the survey. For each ecosystem Figure 1 includes representative examples of surveys without any track detections and surveys with multiple detections. Note that in the case with multiple detections in Kafue, two prides crossed different roads approximately 8 km of one another, which, under real circumstances, would almost certainly be recorded as different tracks and would have led to a high population estimate.

### 4.1 Assessing model performance: Kafue

If the survey method performed well, we would expect the true population size to be captured by the 95% confidence interval in approximate 95% of the simulations. We would also expect a HRCIW of 50% or lower, indicating the estimate has a reasonable probability of detecting a population decline of 50%. If such a decline occurs in 10 years or 3 generations (whichever is longer), then it would meet the IUCN guideline under the A2 criterion, which is applicable to lions, to classify a population or species as endangered. However, it is evident that neither a capturing of the true population size ~95% of the time nor a HRCIW of <50% occurs with this method, and in fact, estimates do not come anywhere close to meeting these criteria. In Kafue, the 95% confidence interval for regular surveys calculated with the track count CV approach rarely included the true population size, ranging 9.6%–20.9% of simulations at 20%–100% survey intensity. The confidence interval using the traditional CV approach captured the true population size in 51.4%–98.2% of simulations at 20%–100% survey intensity. For replicated surveys, the confidence interval calculated with the track count CV approach captured the true population size in 4.4%–17.7% of simulations at 20%–100% intensity, and when all duplicates were removed, this improved to 19.5%–50.3%. For the traditional CV approach, these values were 93.9%–100% and 92.9%–100%, respectively. The HRCIW for surveys with the track count CV approach varied between 10.9% for surveys where all roads were surveyed five times and only duplicates <1,000 m were removed, and 28.4% for surveys where 20% of the roads were surveyed once and only duplicates <1,000 m were removed. The HRCIW for surveys with the traditional CV approach varied between 93.3% for surveys where 40% of the roads were surveyed five times and only duplicates <1,000 m were removed, and 398.3% for surveys where 80% of the roads were surveyed once and only duplicates <1,000 m were removed. Table 4 shows the minimum and maximum percentages of how often the true population size was captured within the 95% confidence interval of the population estimate, the survey types and if all duplicates (not just the tracks of the same prides <1,000 m from each other) have been removed, the survey intensity, and CV approach used. Results from the traditional CV approach are dark shaded, while results from the track CV approach are light shaded. Additionally, it shows the minimum and maximum HRCIW for both methods (in bold). Full results are presented in Appendix S3.

**Table 4:** Summary of results for all modeled scenarios in Kafue. Minimum and maximum percentages of how often the true population was captured in the 95% confidence interval are shown (the traditional CV approach dark shaded, the track count CV approach light shaded), as well as the minimum and maximum HRCIW (in bold). Full results are presented in Appendix S3

Site	Survey type	All duplicates removed	Intensity	CV approach	% true pop captured in 95% CI	HRCIW
Kafue	Regular	No	20	Track count	**9.6%**	**28.4%**
Kafue	Regular	No	80	Track count	**20.9%**	22.2%
Kafue	Regular	No	20	Traditional	**51.4%**	198.9%
Kafue	Regular	No	80	Traditional	96.0%	**398.3%**
Kafue	Regular	No	100	Traditional	**98.2%**	353.3%
Kafue	Replicated	No	20	Track count	**16.1%**	20.0%
Kafue	Replicated	No	80	Track count	**4.7%**	11.2%
Kafue	Replicated	No	100	Track count	5.3%	**10.9%**
Kafue	Replicated	No	20	Traditional	**94.4%**	94.2%
Kafue	Replicated	No	40	Traditional	99.3%	**93.9%**
Kafue	Replicated	No	60	Traditional	**100.0%**	94.3%
Kafue	Replicated	No	80	Traditional	**100.0%**	110.3%
Kafue	Replicated	No	100	Traditional	**100.0%**	108.2%
Kafue	Replicated	Yes	40	Track count	**20.0%**	16.5%
Kafue	Replicated	Yes	100	Track count	**49.8%**	12.9%
Kafue	Replicated	Yes	20	Traditional	**93.6%**	98.7%
Kafue	Replicated	Yes	80	Traditional	**100.0%**	128.0%
Kafue	Replicated	Yes	100	Traditional	**100.0%**	123.2%

Abbreviations: CV, coefficient of variance; HRCIW, half relative confidence interval width.

### 4.2 Assessing model performance: Hwange

In Hwange, the confidence interval calculated with the track count CV approach again rarely included the true population size, varying between 19.7% and 22.4% of simulations for regular surveys at 20%–100% intensity, while the traditional CV approach captured the true population size between 62.9% and 93.1% of simulations. For replicated surveys, the confidence interval calculated with the track count CV approach captured the true population size in 19.2%–25.8% of simulations at 20%–100% intensity, and when all duplicates were removed, this shifted to 0%–37.1%. For the traditional CV approach, these values were 94.4%–100% and 92.4%–99.3% of simulations, respectively. It is interesting to note that for surveys in Hwange, with the CI calculated with the track count CV approach, higher intensities did not necessarily capture the true population more often. The HRCIW for surveys with the track count CV approach varied between 9.4% for surveys where all roads were surveyed five times and only duplicates <1,000 m were removed, and 24.4% for surveys where 20% of the roads were surveyed once and only duplicates <1,000 m were removed. The HRCIW for surveys with the traditional CV approach varied between 88.9% for surveys where 40% of the roads were surveyed five times and only duplicates <1,000 m were removed, and 502.0% for surveys where 80% of the roads were surveyed once and only duplicates <1,000 m were removed. Table 5 shows the same figures as Table 4, but now for Hwange.

**Table 5:** Summary of results for all modeled scenarios in Hwange. Minimum and maximum percentages of how often the true population was captured in the 95% confidence interval are shown (the traditional CV approach dark shaded, the track count CV approach light shaded), as well as the minimum and maximum HRCIW (in bold). Full results are presented in Appendix S3

Site	Survey type	All duplicates removed	Intensity	CV approach	% true pop captured in 95% CI	HRCIW
Hwange	Regular	No	20	Track count	20.4%	**24.4%**
Hwange	Regular	No	60	Track count	**19.7%**	19.4%
Hwange	Regular	No	80	Track count	**22.4%**	18.3%
Hwange	Regular	No	20	Traditional	**62.9%**	210.0%
Hwange	Regular	No	80	Traditional	92.3%	**502.0%**
Hwange	Regular	No	100	Traditional	**93.1%**	419.8%
Hwange	Replicated	No	20	Track count	**19.2%**	16.2%
Hwange	Replicated	No	100	Track count	**25.8%**	**9.4%**
Hwange	Replicated	No	20	Traditional	**94.4%**	91.2%
Hwange	Replicated	No	40	Traditional	99.3%	**88.9%**
Hwange	Replicated	No	80	Traditional	**100.0%**	127.8%
Hwange	Replicated	No	100	Traditional	**99.8%**	122.0%
Hwange	Replicated	Yes	40	Track count	**37.1%**	13.8%
Hwange	Replicated	Yes	100	Track count	**0.0%**	12.0%
Hwange	Replicated	Yes	20	Traditional	**92.4%**	96.3%
Hwange	Replicated	Yes	100	Traditional	**99.3%**	150.5%

Abbreviations: CV, coefficient of variance; HRCIW, half relative confidence interval width.

**Figure 2:**
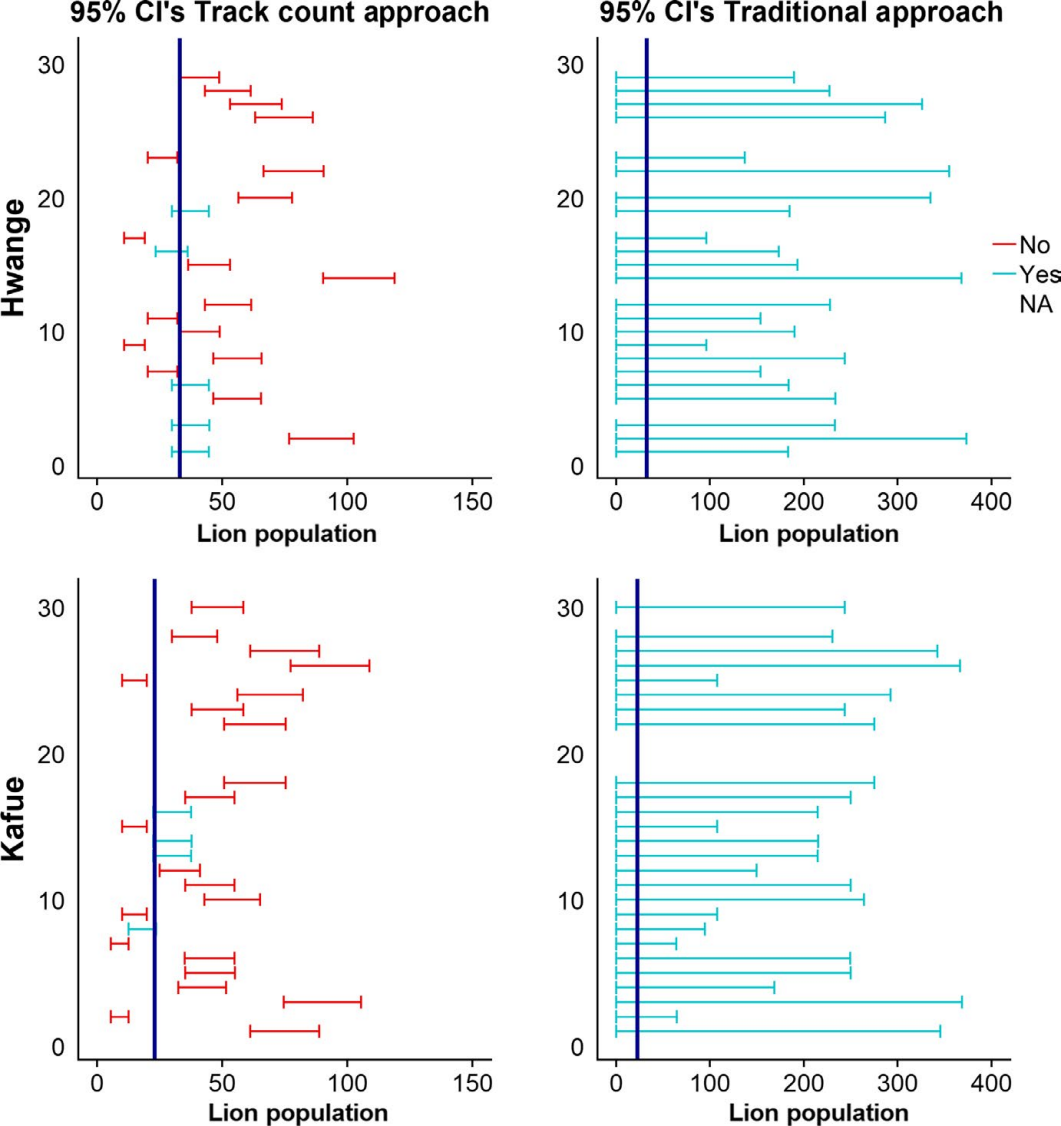
95% confidence intervals for the track count CV approach and the traditional CV approach for regular surveys with a 60% intensity for both Kafue and Hwange. The dark blue vertical line represents the true population size. Missing confidence intervals are cases where the confidence interval could not be calculated because no lions were detected. The track count CV approach often did not contain the true population size, and bias in estimates was not consistent, while the traditional CV approach contained the true population size but with confidence intervals too broad to be biologically meaningful

Figure 2 shows the 95% confidence intervals of population estimates (horizontal lines) and the true population size (vertical line) for a random draw of 30 regular surveys at 60% intensity, for both Kafue and Hwange, with the 95% confidence intervals calculated using CV's following both approaches. This plot reveals an error in the calculation of confidence limits in the track count CV approach (see Section 6 for more detail) that causes precision to be overestimated and thus produces confidence intervals that typically do not include the true population size and the bias of the estimates being inconsistent. The method frequently produced population estimates that differed more than fivefold, despite no change in the true population size. The traditional CV approach produced confidence limits that nearly always contained the true population size, but these confidence intervals were far too wide to describe population trends in a useful manner, as they almost always ranged from 0 to values much greater than the true population size.

## 5 PRECISION

In Kafue, the HRCIW for the method of track count CV approach ranged between 20.6% and 28.4% for regular surveys. For replicated surveys with duplicates, this was 10.9%–20.0% and for replicated surveys without duplicates 13.0%–21.4%. In Hwange, these numbers ranged between 17.2% and 25.6% for regular surveys and between 9.4%–16.6% and 12.0%–18.0% for replicated surveys with and without duplicates, respectively. It should be noted that these intervals frequently did not include the true population size.

In Kafue, the HRCIW for the traditional CV approach ranged between 199.0% and 398.3% for regular surveys and between 94.5%–110.0% and 99.1%–127.9% for replicated surveys with and without duplicates, respectively. In Hwange, these numbers, in their respective order, were 213.1%–520.4%, 91.5%–133.2%, and 98.3%–168.3%. The differences in width of the relative confidence intervals are also shown in Figure 2, with one method failing to capture the true population size most of the time and the other method capturing it but with extremely large confidence intervals.

## 6 DISCUSSION

### 6.1 Track counts do not provide reliable estimates to monitor populations

In realistic simulations based on two lion populations of known size, track surveys either produced confidence limits that did not include the true population size or yielded wide confidence intervals that usually included zero. In either case, the results did not provide estimates of lion population size that could be used to effectively guide conservation and management. The track count CV approach and the traditional CV approach are both likely to yield spurious inferences that a population is increasing or decreasing or that one ecosystem holds a considerably higher or lower population density than another. The traditional CV approach produced confidence intervals so broad that large positive or negative changes in density (or differences in density between ecosystems) would go undetected. The track count CV approach produced confidence intervals that rarely included the true population size.

The reason that the track count CV approach often failed to include the true population size within the confidence interval can be explained as follows. This method originally used a regression with data from several well‐studied populations, including from Hwange National Park, to parameterize a linear equation relating the density of tracks (*t_i_*) to the density of carnivores (*x_i_*).

(1) $$t_{i}=\hat{\beta }x_{i}+\hat{\alpha }$$

The method of using regressions like Equation 1 to convert track counts to population estimates has been widely applied, for example with lions on sandy soils, for which Funston et al. (2010) provide the equation $t_{i}=3.30x_{i}-0.32$. In regressing track density on population density to produce this equation, it was assumed that estimates of population size were equal to the true population size, with no error. This assumption is apparent in two ways. First, Funston et al. (2010) stated that because population estimates “came from long‐term studies of radio‐collared individuals, we do not provide confidence limits.” Second, the estimates of population density were treated as an independent variable measured without error in their Figure 2, which shows the regression of track density on population density.

Ignoring variance in estimates of population size is problematic when developing a method to estimate population size from its relationship to another variable (tracks, in this case). The variance associated with the original population estimates was not used in the regression equation to convert track counts to population estimates, and consequently, the precision of population estimates using this method is overstated: Confidence limits are affected by the sampling variance of tracks, but not by the sampling variance associated with a specific track density, because it was assumed to be zero. Funston et al. (2010) did not describe the methods used to produce the population estimates included in their regression, beyond noting that they came from long‐term studies of radio‐collared individuals. However, the variance of such population estimates for large carnivore populations is appreciable, even with intensive, long‐term monitoring using radio‐collars (e.g., see M'Soka, Creel, Becker, & Droge, 2016; Mweetwa et al., 2018; Rosenblatt et al., 2014). This uncertainty is too large to ignore, but it is not addressed by surveys that use the methods of Funston et al. (2010) to convert estimates of track density to estimates of population density. Consequently, the precision of these population estimates is overstated. As a result, the associated confidence intervals often do not contain the true population size. Additionally, the bias in the estimates is inconsistent, making it of no value for monitoring population trends.

Compounding this problem, track surveys use $\hat{\alpha }$ and $\hat{\beta }$ from Equation 1 to convert the confidence limits for track density into confidence limits for carnivore density. This conversion (incorrectly) treats the relationship between track and carnivores as if it was a back‐transformation, rather than (correctly) applying the equation for the variance of a linear function. When a variable (e.g., lion density) is a linear function of other variables (e.g., track density), then the mean of the linear function is equal to the linear function applied to the constituent means, but the variance of the linear function is not equal to the linear function applied to the constituent variances. To clarify, if *X*_1_, *X*_2_, … *X_n_* are independent random variables with means *μ*_1_, *μ*_2_, … *μ_n_* and variances $\sigma_{1}^{2},\sigma_{2}^{2},\cdots \sigma_{n}^{2}$ and *Y* is a linear function of these variables

(2) $$Y=\sum_{i=1}^{n}a_{i}X_{i}$$

with constants *a*_1_, *a*_2_, … *a_n_*_,_ then the mean of *Y* is

(3) $$\mu_{Y}=\sum_{i=1}^{n}a_{i}\mu_{i}$$

and the variance of *Y* is

(4) $$\sigma_{Y}^{2}=\sum_{i=1}^{n}a_{i}^{2}\sigma_{i}^{2}.$$

Critically, the constant relating *Y* (lion density) to *X* (track density) is squared in Equation 4. By treating the conversion of variance as a simple linear back‐transformation, many recent carnivore assessments have compounded the overstatement of precision by a factor of 3.30 (i.e., the slope $\hat{\beta }$ of Equation 1 estimated by Funston et al. (2010), which becomes *a_i_* in Equation 4). After addressing these problems, confidence limits on estimates from track surveys would often be >5 times wider than stated and be similar to the confidence intervals calculated according to the conventional method of calculating CV's. A clear recognition of this uncertainty is needed for valid assessments of large carnivore population sizes, trends, and the success or failure of efforts to conserve them.

We also reported the percentage of times that not a single set of lion tracks was encountered. In both Kafue (1.8%) and Hwange (6.9%), there were surveys, where 100% of roads were surveyed once and no tracks were encountered, though it should be recognized that these values were for simulated densities, lower than the true densities in these ecosystems. Nonetheless, the percentage of surveys that detect no lion tracks is expected to be appreciable at low but realistic lion densities in areas with few roads, which are precisely the areas where better information about lion population is needed. In Angola, for example, countrywide estimates were adjusted from >1,000 to <50 largely based on track surveys which detected very few lion tracks (Funston et al., 2017; Overton, Fernandes, Elizalde, Groom, & Funston, 2017). Anderson (2001) pointed out that index calibration survey methods assume that detection probability is constant. Following this logic, the number of detections of sign should change in proportion to changes in the population, or survey effort. Thus, in our simulations, survey intensities of 80% should have twice as many observations as survey intensities of 40%. Indeed, in Kafue for example, the mean number of tracks detected in regular surveys goes from 9.9 to 18.3 in surveys with 40% and 80% respectively. However, the minimum number of tracks detected at both intensities is 0 and the maximum number of tracks detected during surveys was 70 and 78, respectively. Despite a doubling of effort, the ranges for the number of detected tracks overlap almost completely. This substantial variation in detection, violates the critical ICS assumption of constant detection probability, and strongly limits the inferences that can be drawn from index calibration methods for low‐density, wide‐ranging species. The sources of variation in the number of intersections between tracks of wide‐ranging and low‐density animals and survey transects (speed, distance, and direction of movement of the animals which can be influenced by prey density, feeding state, weather, moon phase among many other things) apply over large spatial scales and thus, in reality, are large. Our simulations indeed show that there is considerable variation in detection with no change in underlying population density. In theory, this variation could be controlled if many observations are collected; however, since these animals occur at low densities that is not practical*,* as even when surveys were replicated 80 times (resulting in survey lengths of tens of thousands of kilometers), this variation still did not increase the power of track surveys to detect population trend to acceptable levels.

### 6.2 What is considered to be a “good” population estimate?

Numerous authors (Elliot & Gopalaswamy, 2016; Funston et al., 2010; Kane, Morin, & Kelly, 2015; López‐Bao et al., 2018; Reynolds, Thompson, & Russell, 2011; Seavy & Reynolds, 2007) emphasize the need for reliable, unbiased, and precise population estimates for animals. However, only Reynolds et al. (2011) quantifies what would be sufficient precision, and they do so using the well‐developed IUCN guidelines criteria for the classification of the status of species or populations. Lions are currently listed by the IUCN as vulnerable under criterion A2 (Bauer, Packer, Funston, Henschel, & Nowell, 2016). Under the A2 criterion, a species will be classified as “vulnerable” if there was a 30% decline in the population in the past 10 years or 3 generations (whichever is longer) and would be classified as “endangered” if there was a 50% decline. For lions, the best available data led to an estimate of 25,105 lions in 2018, down from 33,292 lions in 2005 (IUCN SSC Cat Specialist Group, 2018). Measures of precision do not accompany these estimates, but in the report it is noted that “Many of the estimates we present have very large confidence intervals, and for many the precision is not even known.” One recommendation made in the Guidelines for Using the IUCN Red List Categories and Criteria (IUCN, 2017) is that a precautionary attitude should be adopted, using plausible lower bounds, rather than best estimates. Since no uncertainty is reported this is not possible, and it leaves room for debate if lions should be listed as “near threatened,” “vulnerable,” or “endangered.” Even for an iconic and economically valuable species, like the lion, published population estimates often do not meet the standard of the IUCN guidelines for assessing the status of the species; these standards currently require the power to infer a 30%, or larger, decline. This would be the equivalent of a HRCIW of 30%, yet only a few population estimates, from even fewer populations, and all from long‐term intensive studies meet those standards (Loveridge, Valeix, Davidson, et al., 2016; Mweetwa et al., 2018; Rich et al., 2019; Rosenblatt et al., 2014). The argument for the need for enough statistical power to detect trends in populations is not new. Macdonald, Mace, & Rushton noted in 1998 (summarized in Macdonald, Mace, & Rushton, 2000) that there is a risk with many monitoring schemes, that all the grueling effort of fieldworkers is wasted because there is so much “noise” in the data that the statistical power is too low to detect changes in a species' numbers.

We recommend that monitoring of large carnivores like the lion use manageable‐sized and representative areas, with methods that can detect a 30% change in any 10‐year period, according to the IUCN guidelines. This criterion could possibly be relaxed for application in the field by protected area managers, by using an 80% confidence interval rather than a 95% confidence interval and tightening the time span to 5 years or 1 generation time. A usefully precise estimate from a well‐chosen representative area, as big as could logistically be covered with the resources available, is a logical basis for the management of the wider landscape; as with all sampling designs, careful consideration about the broader area that a study site represents could allow inferences to guide management over a larger area than the study site itself.

Populations where human encroachment and prey densities are not changing dramatically are not likely to experience dramatic population declines within short times; therefore, surveys to detect population changes in such areas should consider periods that realistically reflect lion demography. Most population estimates consider subadults and adult lions; cubs are generally considered subadults at 18–24 months, but this is not consistent between studies. If there is no continuous monitoring program in place, it would be advisable to invest in more intensive or longer (or both) surveys bi‐annually, or tri‐annually, versus annual surveys or short one‐off surveys. Elliot and Gopalaswamy (2016) showed that in a high lion density area, and where lions are easily sighted, it is possible to get estimates which fall well within the IUCN precision requirements with a 3‐month intensive spatial mark–recapture approach. For areas with lower lion densities, or where lions are more cryptic, a longer period and/or the aid of camera traps, from which lions could also be individually identified, is more advisable. One has to consider assumptions of closure when studying animals over longer periods or choose approaches which can deal with closure assumptions over longer periods like robust design. Furthermore, we recommend adding the HRCIW to population or density estimates as an easy to calculate and easy to interpret figure evaluating the estimate's power to detect population changes.

### 6.3 ICS estimate do not provide “good” population estimates

The recently published Guidelines for the Conservation of Lions in Africa (IUCN SSC Cat Specialist Group, 2018) includes a chapter on the monitoring of lion populations. It focuses heavily two ICS methods, the track count survey methods and the call‐up survey method (for a more detailed explanation about the call‐up survey method see Ogutu and Dublin (1998) and Ferreira and Funston (2010)). In conducting call‐up surveys, many authors found the response rate, and thus the detection probability, of lions to vary with different factors—such as complete groups of lions responding or none at all (Brink et al., 2013), the distance between the lion and the speaker and speaker placement relative to the core area of a pride's territory, age, sex, presence of resident males, group size, and whether the lions possessed a carcass (Whitman, 2006) and the presence of cubs (Mills et al., 2001; Ogutu & Dublin, 1998; Whitman, 2006). Arguably, the vegetation type, ruggedness of the terrain and wind conditions play a role too in how far the sound is carried and from how far lions could be attracted. However, the method only distinguishes in the response rate between prides with and without cubs (Ferreira & Funston, 2010; IUCN SSC Cat Specialist Group, 2018). Detection probability is assumed to be constant within these two classes, but this assumption is known to be false, violating a critical assumption of ICS. The complexity of using the call‐up method is further increased by the recommendation to calibrate a site‐specific response rate by testing with >20 groups of lions, something which is very rarely achieved. For example, in Niassa Reserve, where some of the most extensive call‐up surveys have been conducted—with up to 153 call‐up stations per survey conducted within a year—only 16 prides in total responded (Begg, Miller, & Begg, 2018). Belant et al. (2016) found that lions habituate to call‐up sounds very quickly and that temporal and spatial variation of broadcasted sound did not reduce this habituation (Belant et al., 2017), which would imply that areas used for calibration cannot subsequently be surveyed. We did not evaluate the call‐up method here. However, the similarities in the assumptions between both methods, assuming constant detection probabilities while ignoring many sources contributing to variation in detection probabilities, together with the recommendation of its use in the Guidelines for the Conservation of Lions in Africa (IUCN SSC Cat Specialist Group, 2018) leads us to caution against the use of the call‐up method as well.

Johnson (2008) noted that a variety of methods have been developed to improve ICS. However, these methods either focus on improving or better estimating detection probability, and in our simulation, we assumed perfect detection every time that tracks intersect a survey. Repeated counts could be used to improve ICS in various ways. One approach is to take the maximum count of tracks as an estimate of minimum population size. Such a method is often used in point counts for birds, for example for estimating grouse through counting at leks (Walsh, White, Remington, & Bowden, 2004). However, these authors acknowledge that the probability of detecting an individual must be estimated (by mark–recapture) to relate lek counts to population size (Anderson, 2003). Moreover, in our simulations the maximum count was often greater than the population size, except in cases where all duplicates were removed, which would typically be impossible for a field study. This problem is illustrated in Figure 1, where several prides are recorded more than once, crossing different roads at distances up to ~8 km apart.

The track count method has also been used to determine occupancy rather than density (Funston et al., 2017; Henschel et al., 2016; Midlane et al., 2014), and it is likely that it performs better in this context. However, given the large variation in detection between different surveys, revealed by our simulations, caution should be used when using track count data for occupancy analyses and a simulation study of this method would be of value.

Overall, we conclude that index calibration survey methods are not effective for population monitoring of wide‐ranging low‐density species like lions. While inexpensive methods that can rapidly be applied across large areas would be of great value, our simulations show that the track count methods currently used to monitor a range of African large carnivores produce population estimates with overstated precision (leading to erroneous inferences about population trends or differences between ecosystems) or with power that is too low to guide management. To date, only resource and time intensive long‐term studies with individual recognition have produced population estimates with precision sufficient to apply IUCN criteria for decisions about the status of lion populations (Loveridge, Hemson, Davidson, & Macdonald, 2010; Loveridge, Valeix, Davidson, et al., 2016; Mweetwa et al., 2018; Rosenblatt et al., 2014). In some cases, relatively short intensive studies have yielded precise estimates of lion density using spatially explicit capture–recapture (SECR) techniques, in areas with a relatively high population density, with animals that are easily approachable by vehicle (Elliot & Gopalaswamy, 2016).

Long‐term intensive studies are expensive and challenging to maintain but population dynamics are rarely the focal point of published studies. Regularly publishing demographic data from these studies would provide critical data for conservation and management purposes, but might not be a scientific priority, which often leads to rigorous population trend data not being publicly available for many well‐studied species of concern. The exception to this rule is the 5‐yearly large scale monitoring survey done for tigers throughout India (Jhala et al., 2019), even though this survey is done in a single country, it is done across many populations, in a consistent manner, which could set an example for monitoring of populations of several large carnivore species elsewhere across countries and populations. Individually based (spatial) capture–recapture methods benefitting from advances in genetic approaches like pedigree estimation (Creel & Rosenblatt, 2013; Spitzer et al., 2016) and improvement in quality of camera trap images hold some promise to reduce the cost of long‐term capture–recapture studies and additional research and development into these methods is strongly recommended. We also echo the call that concludes the lion monitoring chapter in the Guidelines for the Conservation of Lions in Africa (IUCN SSC Cat Specialist Group, 2018) that there is a need for a more comprehensive overview on the collection of lion data, and other wide‐ranging low‐density species, and that a specific handbook on lion monitoring methods is urgently needed to increase the quality and comparability of lion monitoring throughout its range to ensure the survival of the species in Africa and Asia. Such a handbook could also be a guideline for monitoring practices for other low‐density, wide‐ranging species for which such practices need to be comparable to make wide‐range inferences with data from different studies, populations and countries.

## CONFLICT OF INTEREST

None declared.

## AUTHORS' CONTRIBUTIONS

ED and SC conceived the ideas and designed methodology. MSB, SC, DWM, and AL provided data. ED and LLS designed the simulations. ED and SC led the writing of the manuscript. All authors contributed critically to the drafts and gave final approval for publication.

## Data Availability

Data of roads and lion movements and R scripts to generate random transects and perform simulations are deposited into Dryad and are available at: https://doi.org/10.5061/dryad.37pvmcvfv
