# Supplementary material for: Assessing the performance of index calibration survey methods to monitor populations of wide‐ranging low‐density carnivores
Source: Ecol Evol. 2020 Mar 6;10(7):3276–92. doi: 10.1002/ece3.6065 (PMC7141012; doi:10.1002/ece3.6065)
Supplement: Supplementary file 1 [file ECE3-10-3276-s001.docx]

Appendix I – The construction of random transects

We used shapefiles of the existing road network from both study sites. Each transect set consisted of a number of transects. The accumulative lengths of the transects were set at 20%, 40%, 60%, 80% and 100% of the existing road network as intended survey intensity. As transects were 10km or 5km long, the total length of transects was usually a little less than the intended survey intensity, but for our analysis we used actual transect lengths. Using the sp package (Bivand, Pebesma, & Gomez-Rubio, 2013; Pebesma & Bivand, 2005) in R we first converted the road shapefiles from lines into evenly spaced points 50m apart. A transects were either 5km or 10km long they contained either 100 or 200 points; the threshold to complete transects. To build transect *n*, we randomly selected a point not already assigned to a transect and added this point to transect *n*. Then, a buffer of 55m was placed around this point, and randomly a point not already assigned to a transect, within the buffer, was selected and added to transect *n*. We did this until the number of points assigned to transect *n* met the threshold number for that transect. If there were no unassigned points available within the buffer, all the points assigned to the current transect being built were de-assigned and a new random point was selected to start building another transect.

This method minimized bias by ensuring random starting points of transects, random trajectories of transects along existing roads, and transects of sufficient length. For each study area we assigned ~50% of transects to 5km length and ~50% of transects to 10km length for surveys of 20-60% intensity. The longer 10km transects were created first, and then the 5km transects were created along the remaining roads which sped up the creation of random transects considerably. By increasing the number of randomly spaced transects and randomly creating the 5km transects along roads not covered by the 10km transects we ensured that transects covered the study area well and were sufficiently close together that they did not miss home ranges of lion prides. Thus, we simulated a situation in which the transect sampling design was well-optimized. With higher intensities (>= 60%) transects were rarely more than one transect length from each other. Due to challenges in generating enough transects for surveys at 80% intensity, we randomly created 5km transects at an intensity of 20% and subtracted those from the existing road network to identify the transects. For survey at 100% we used QGIS 3.2 (QGIS Development Team 2018) to cut the road network in segments of maximum 10km lengths. This left many small segments, and we deleted any stretch <500m, resulting in 462.1 km of a total of 467.2 km of the roads being surveyed in Kafue National Park and 592.5 km of a total of 608.9 km of the roads being surveyed in Hwange respectively. With these procedures, the exact sampling intensity was typically slightly less than the nominal intensity, so actual (rather than intended) transect lengths were used in all analyses. We added a buffer of 25 meters to all transects to increase the possibility of intersection with lion tracks and decrease the possibility of missing intersections because of locational errors of either the roads or lion trajectories. A graphical diagram of the construction of transects can be found in Appendix I.
