# Supplementary material for: Assessing the performance of index calibration survey methods to monitor populations of wide‐ranging low‐density carnivores
Source: Ecol Evol. 2020 Mar 6;10(7):3276–92. doi: 10.1002/ece3.6065 (PMC7141012; doi:10.1002/ece3.6065)

Appendix II

A step-by-step graphical overview of the construction of random transects from existing roads.


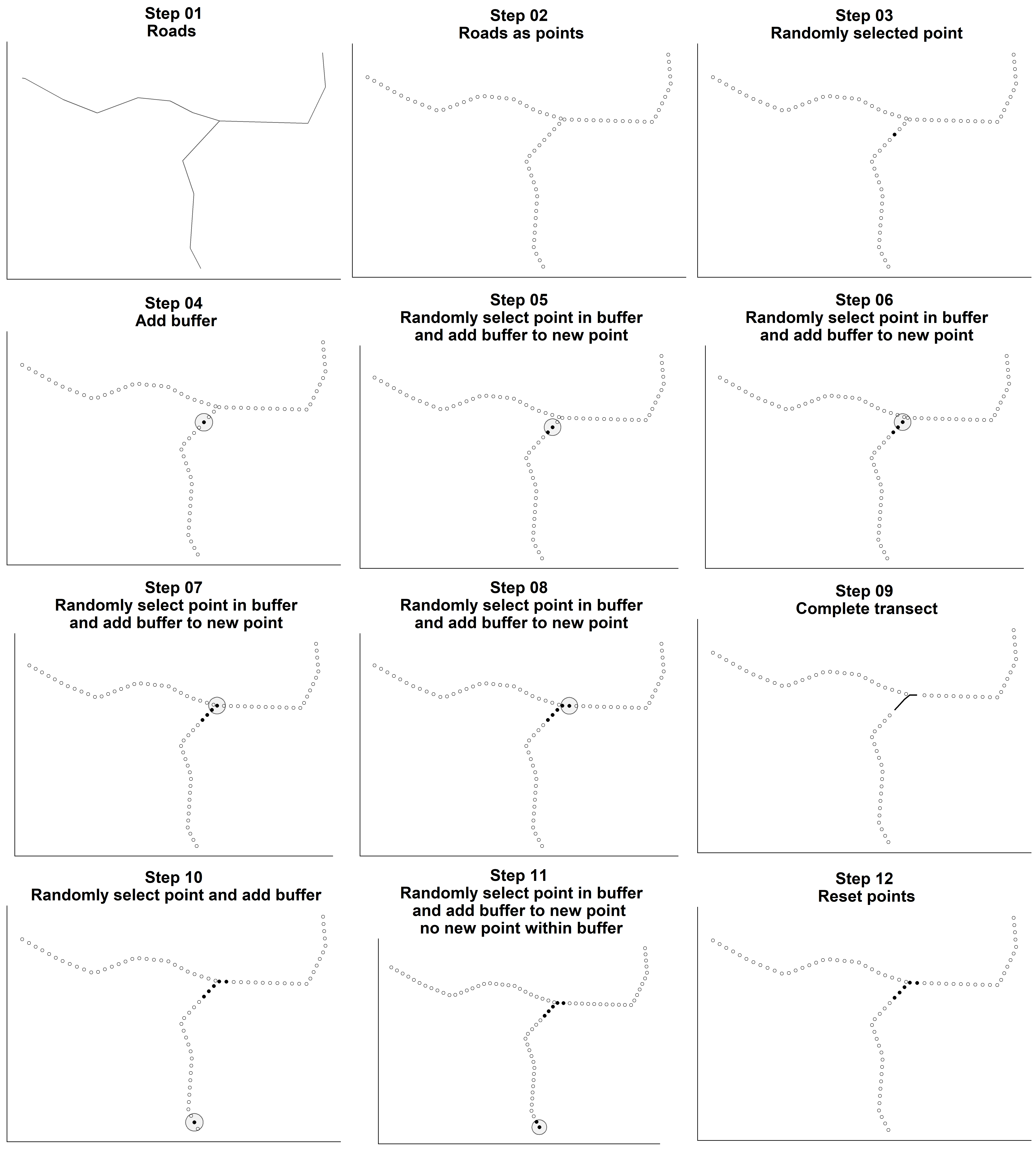

Supplement: Supplementary file 2 [file ECE3-10-3276-s002.docx]
