# Supplementary material for: Assessing the performance of index calibration survey methods to monitor populations of wide‐ranging low‐density carnivores
Source: Ecol Evol. 2020 Mar 6;10(7):3276–92. doi: 10.1002/ece3.6065 (PMC7141012; doi:10.1002/ece3.6065)
Supplement: Supplementary file 3 [file ECE3-10-3276-s003.docx]

Full table 4: Full results of model performance for all modeled scenarios in Kafue, minimum and maximum percentages of how often the true population was captured in the confidence interval are displayed in **bold**.

| **Site** | **Survey type** | **All duplicates removed** | **Intensity** | **CV approach** | **% true pop captured in 95% CI** | **HRCIW** |
| --- | --- | --- | --- | --- | --- | --- |
| Kafue | Regular | No | 20 | Track count | **9.6%** | 28.4% |
| Kafue | Regular | No | 40 | Track count | 11.4% | 26.2% |
| Kafue | Regular | No | 60 | Track count | 20.2% | 23.8% |
| Kafue | Regular | No | 80 | Track count | **20.9%** | 22.2% |
| Kafue | Regular | No | 100 | Track count | 15.8% | 20.6% |
| Kafue | Regular | No | 20 | Traditional | **51.4%** | 198.9% |
| Kafue | Regular | No | 40 | Traditional | 78.4% | 243.4% |
| Kafue | Regular | No | 60 | Traditional | 91.4% | 268.8% |
| Kafue | Regular | No | 80 | Traditional | 96.0% | 398.3% |
| Kafue | Regular | No | 100 | Traditional | **98.2%** | 353.3% |
| Kafue | Replicated | No | 20 | Track count | **16.1%** | 20.0% |
| Kafue | Replicated | No | 40 | Track count | 9.2% | 15.0% |
| Kafue | Replicated | No | 60 | Track count | 6.4% | 12.7% |
| Kafue | Replicated | No | 80 | Track count | **4.7%** | 11.2% |
| Kafue | Replicated | No | 100 | Track count | 5.3% | 10.9% |
| Kafue | Replicated | No | 20 | Traditional | **94.4%** | 94.2% |
| Kafue | Replicated | No | 40 | Traditional | 99.3% | 93.9% |
| Kafue | Replicated | No | 60 | Traditional | **100.0%** | 94.3% |
| Kafue | Replicated | No | 80 | Traditional | **100.0%** | 110.3% |
| Kafue | Replicated | No | 100 | Traditional | **100.0%** | 108.2% |
| Kafue | Replicated | Yes | 20 | Track count | 21.7% | 21.4% |
| Kafue | Replicated | Yes | 40 | Track count | **20.0%** | 16.5% |
| Kafue | Replicated | Yes | 60 | Track count | 22.0% | 14.3% |
| Kafue | Replicated | Yes | 80 | Track count | 43.3% | 13.5% |
| Kafue | Replicated | Yes | 100 | Track count | **49.8%** | 12.9% |
| Kafue | Replicated | Yes | 20 | Traditional | **93.6%** | 98.7% |
| Kafue | Replicated | Yes | 40 | Traditional | 99.0% | 99.9% |
| Kafue | Replicated | Yes | 60 | Traditional | 99.9% | 102.0% |
| Kafue | Replicated | Yes | 80 | Traditional | **100.0%** | 128.0% |
| Kafue | Replicated | Yes | 100 | Traditional | **100.0%** | 123.2% |

Full table 5: The full results of model performance for all modeled scenarios in Hwange, minimum and maximum percentages of how often the true population was captured in the confidence interval are displayed in **bold**.

| **Site** | **Survey type** | **All duplicates removed** | **Intensity** | **CV approach** | **% true pop captured in 95% CI** | **HRCIW** |
| --- | --- | --- | --- | --- | --- | --- |
| Hwange | Regular | No | 20 | Track count | 20.4% | **24.4%** |
| Hwange | Regular | No | 40 | Track count | 19.2% | 21.5% |
| Hwange | Regular | No | 60 | Track count | **19.7%** | 19.4% |
| Hwange | Regular | No | 80 | Track count | **22.4%** | 18.3% |
| Hwange | Regular | No | 100 | Track count | 26.2% | 17.2% |
| Hwange | Regular | No | 20 | Traditional | **62.9%** | 210.0% |
| Hwange | Regular | No | 40 | Traditional | 84.0% | 242.4% |
| Hwange | Regular | No | 60 | Traditional | 90.1% | 256.1% |
| Hwange | Regular | No | 80 | Traditional | 92.3% | **502.0%** |
| Hwange | Regular | No | 100 | Traditional | **93.1%** | 419.8% |
| Hwange | Replicated | No | 20 | Track count | **19.2%** | 16.2% |
| Hwange | Replicated | No | 40 | Track count | 15.7% | 12.0% |
| Hwange | Replicated | No | 60 | Track count | 17.0% | 10.6% |
| Hwange | Replicated | No | 80 | Track count | 18.0% | 9.6% |
| Hwange | Replicated | No | 100 | Track count | **25.8%** | **9.4%** |
| Hwange | Replicated | No | 20 | Traditional | **94.4%** | 91.2% |
| Hwange | Replicated | No | 40 | Traditional | 99.3% | **88.9%** |
| Hwange | Replicated | No | 60 | Traditional | 99.7% | 90.1% |
| Hwange | Replicated | No | 80 | Traditional | **100.0%** | 127.8% |
| Hwange | Replicated | No | 100 | Traditional | **99.8%** | 122.0% |
| Hwange | Replicated | Yes | 20 | Track count | 27.0% | 17.5% |
| Hwange | Replicated | Yes | 40 | Track count | **37.1%** | 13.8% |
| Hwange | Replicated | Yes | 60 | Track count | 29.3% | 12.7% |
| Hwange | Replicated | Yes | 80 | Track count | 0.3% | 12.3% |
| Hwange | Replicated | Yes | 100 | Track count | **0.0%** | 12.0% |
| Hwange | Replicated | Yes | 20 | Traditional | **92.4%** | 96.3% |
| Hwange | Replicated | Yes | 40 | Traditional | 98.2% | 97.4% |
| Hwange | Replicated | Yes | 60 | Traditional | 98.1% | 101.5% |
| Hwange | Replicated | Yes | 80 | Traditional | 99.9% | 159.3% |
| Hwange | Replicated | Yes | 100 | Traditional | **99.3%** | 150.5% |
